# Motion perception in touch: resolving contradictory findings by varying probabilities of different trial types

**DOI:** 10.1007/s00426-023-01849-1

**Published:** 2023-06-27

**Authors:** Simon Merz, Christian Frings, Charles Spence

**Affiliations:** 1 Department of Psychology, Cognitive Psychology, University of Trier, Universitätsring 15, 54286 Trier, Germany; 2 Department of Experimental Psychology, University of Oxford, Oxford, UK

## Abstract

Representational momentum describes the typical overestimation of the final location of a moving stimulus in the direction of stimulus motion. While systematically observed in different sensory modalities, especially vision and audition, in touch, empirical findings indicate a mixed pattern of results, with some published studies suggesting the existence of the phenomenon, while others do not. In the present study, one possible moderating variable, the relative probabilities of different trial types, was explored in an attempt to resolve the seemingly contradictory findings in the literature. In some studies, only consistently moving target stimuli were presented and no representational momentum was observed, while other studies have included inconsistently moving target stimuli in the same experimental block, and observed representational momentum. Therefore, the present study was designed to systematically compare the localization of consistent target motion stimuli across two experimental blocks, for which either only consistent motion trials were presented, or else mixed with inconsistent target motion trials. The results indicate a strong influence of variations in the probability of different trial types on the occurrence of representational momentum. That is, representational momentum only occurred when both trial types (inconsistent and consistent target motion) were presented within one experimental block. The results are discussed in light of recent theoretical advancements in the literature, namely the speed prior account of motion perception.

The localization of moving objects is a crucially important task for our daily interaction with the external world. Representational Momentum (RM) describes a typical localization bias in which the perceived final position of a dynamically-changing, moving object is systematically overestimated in the direction of motion (Freyd & Finke, 1984). This overestimation, often described as a forward shift, has by now been observed in numerous studies with different experimental features (see Hubbard, 2005, 2018, for extensive reviews). Interestingly, while most studies have explored RM in the visual modality, the evidence suggests that this bias also occurs in audition (Getzmann et al., 2004; Schmiedchen et al., 2013). More recently, the phenomenon has been explored in the tactile modality as well, yet, the observed pattern of results for tactile stimuli remain somewhat inconclusive. While some evidence suggests the existence of RM in touch (e.g., Merz et al., 2019a, 2019b, 2022), other studies have failed to observe a tactile analogue of the phenomenon (e.g., Macauda et al., 2018; see, relatedly, Whitsel et al., 1986). This is surprising insofar as the studies have used fairly similar stimulus speeds (about 6 to 7 cm/s), with stimulus speed being a central moderating influence on the RM phenomenon (see Hubbard, 2005, 2018; Merz et al., 2022, for extensive discussions).

Upon closer inspection of the studies, one central difference between those studies investigating RM in the tactile modality that have observed the forward shift (e.g., Merz et al., 2019a, 2019b, 2022) and those that have not (e.g., Macauda et al., 2018; Whitsel et al., 1986) was the probability of different trial types. With trial type probability, we describe the composition of different trial types which are included in one experimental block. To be more precise, in those studies where RM was not observed, only consistent motion trials (successive presentations of the target in one consistent direction and with one consistent stimulus speed) were presented. Yet, these studies did not include a baseline measure to account for other localization biases, independent from the consistent, directional motion of the stimulus, which have often been shown in the tactile localization literature (e.g., the centering bias; Brooks et al., 2019; Nelson et al., 2019, further influenced by intensity, e.g. Steenbergen et al., 2014; the head orientation biases, Ho & Spence, 2007, anchoring by landmarks such as elbow or wrist, e.g., Cholewiak & Collins, 2003). To account for this shortcoming, Merz and colleagues (Merz et al., 2019a, 2019b, 2020, 2023a) also included inconsistent motion trials within the same experimental block. Yet, it is unclear whether the inclusion of these inconsistent motion trials within the same experimental block in-and-of-itself changed the perception of the consistent motion trials, which is the focus of the present study.

The usage of a baseline condition was argued for not just in touch by the studies of Merz et al. (e.g., Merz et al., 2019b, 2022), but similar arguments and experimental designs have been made in the visual and auditory modalities (see Freyd & Finke, 1984; Getzmann & Lewald, 2007; Getzmann et al., 2004). Yet, what was different in Merz et al.’s studies was that these baseline (inconsistent motion) trials were presented in the same experimental block as the consistent motion trials. This raises the question of whether the inclusion of inconsistent motion trials within one experimental block has influenced the perception for the consistent motion trials. Previously, RM has been shown to be influenced by attentional factors (Hayes & Freyd, 2002) as well as by an observer’s expectations about typical target behaviour (e.g., Reed & Vinson, 1996; Vinson & Reed, 2002; see Hubbard, 2005, 2018; for a detailed discussion). Therefore, the present study was specifically designed to explore the possible influence of varying the probabilities of different trial types within a single experimental block, by comparing the perception of consistent motion trials in two task settings – either presented mixed with inconsistent motion trials, or only consistent motion trials are presented. If an effect were to be observed, this would suggest that the processes underlying RM is dependent on the probability of trial types in which motion is perceived.

The question arises as to why the trial type probabilities might influence motion perception. The processing of moving features such as direction and orientation is already observed early in neural processing, e.g., in the primary cortices (Pei et al., 2011; see Pei & Bensmaia, 2014, for an extensive discussion of the neural underpinnings of tactile motion and its similarities to visual motion processing). That can, however, be modulated by feedback from higher order brain areas as RM has been shown to be influenced by expectation (Reed & Vinson, 1996; Vinson & Reed, 2002). So why might the task setting, more precisely, the inclusion of inconsistent motion trials, influence the occurrence of RM? One possibility here can be derived from the speed prior account (Merz et al., 2022) that has recently been suggested as an explanation for RM (and other motion-related biases). In light of this account, the trial type probability might be one factor to set different speed priors, that is, different expectations about the typical speed presented within the current experimental setting. Therefore, with different task settings inducing different speed expectations, the same stimulus can be perceived differently, as perceived speed is thought to reflect both the actual speed and prior speed expectation. We return to this issue in the Discussion.

In order to investigate the possible influence of including inconsistent motion trials within the same experimental block, the typical RM set-up from many previous studies was used (Merz et al., 2019a, 2019b, 2020, 2022). That is, vibrotactile stimulation was presented to the left forearm, with vibrotactile stimulators (also termed tactors) attached to the back of a touchscreen and then attached to the forearm (for a visualization, see Fig. 1A, B). The timing of events was the typical consistent motion sequence with a 250 ms stimulus duration as well as interstimulus interval (often also termed implied motion; for similar approaches in vision, see Freyd & Finke, 1984; Hubbard & Ruppel, 2014). For each trial, a sequence of three vibrations was presented, and participants had to indicate on the touchscreen the location of the final vibration. Two different sequences were presented to the participants, as customary in previous studies of RM in touch (Merz et al., 2019a, 2019b). For the consistent motion sequence, three tactile stimuli implied consistent directional motion, that is, the stimuli were presented adjacent to each other translating in a consistent direction and with a consistent speed in every trial. For the inconsistent motion sequence, the locations were selected randomly without replacement with the restriction that consistent motion trials never occurred. The resulting target sequence was therefore less predictable in terms of its direction and speed.

**Fig. 1:**
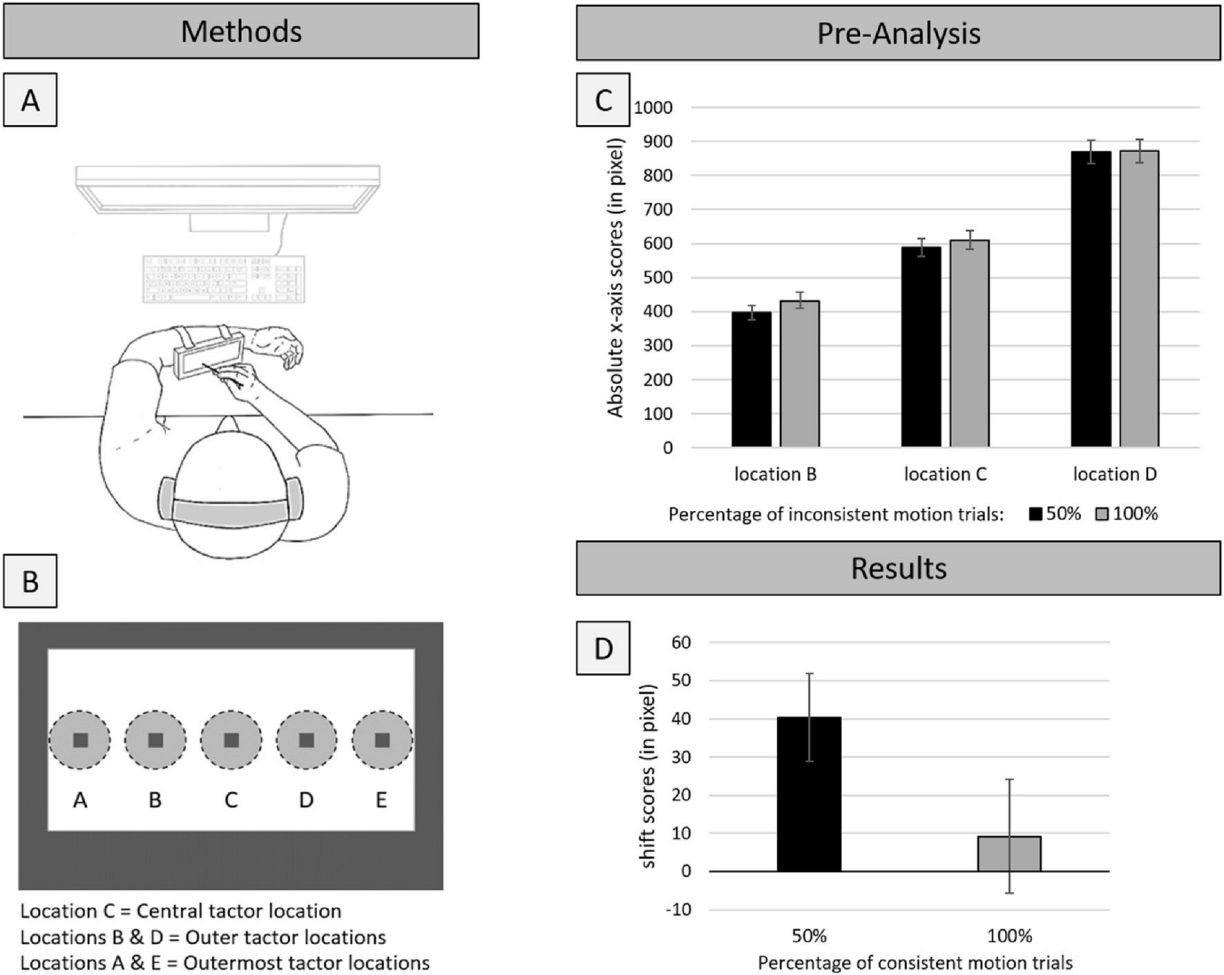
Methods and results of the experiment. Methods: **A** Bird’s-eye view of the experimental set-up including the touchscreen, attached to the participant’s left forearm. **B** Close-up view of the backside of the touchscreen, five tactor are attached to present the vibrotactile stimuli. **C** Depiction of the pre-analysis results of the inconsistent motion trials as a factor of experimental condition and actual target location, with absolute x-axis scores as dependent variable. **D** Depiction of the shift scores (difference between inconsistent and consistent motion trials) as a factor of experimental condition. Error bars represent standard errors of the mean. For more details, see main text

Three different trial type probabilities were realized for each participant: In one experimental block, only the consistent motion trials were presented, in a second, only the inconsistent motion trials were presented, and in a third, both trial types were mixed within one experimental block. The inconsistent motion trials are used as a baseline measure to account for the numerous general localization biases, independent from consistent directional motion, that are well-known in the literature on tactile localization (e.g., the centering bias; Brooks et al., 2019; Nelson et al., 2019; Steenbergen et al., 2014; anchoring by landmarks such as elbow or wrist, e.g., Cholewiak & Collins, 2003; Mancini et al., 2011). Subsequently, the difference between the inconsistent and consistent motion trials is used as the dependent variable, as this difference allows for the pure assessment of any potential influence the consistent direction motion has on tactile localization. For the inconsistent trials, we would not expect any influence of trial type probability and yet we explicitly test any potential influence in a pre-analysis. In a next step, we then compute the difference scores between the consistent and inconsistent motion trials, the representational momentum effect, to analyse any potential influence of variations in the probability of different trial types.

Based on previous results, it was expected that in the mixed condition, the typical forward shift (RM effect) would be observed, as has often been reported previously (Merz et al., 2019a, 2019b). The central question then concerns the presentation condition in which only consistent motion trials are presented. If no RM, or even a backward shift (that is, a shift against the direction of motion), were to be observed in this condition, this would resolve the contrasting findings that have been published in the literature to date, and indicate the varying probability of different trial types as a crucial moderating influence on RM (Macauda et al., 2018; Merz et al., 2019a, 2020).

## Methods

### Participants

Tactile localization biases on their own typically elicit medium to large effect sizes (dz around 0.6), therefore we aimed for at least 26 participants to find a shift at the minimum (α < 0.05; 1 − β > 0.90; power analyses were run with G-Power 3.1.9.2, option ‘means: difference from constant’; Faul et al., 2007). To account for possible drop-outs and to allow for counterbalancing across participants, a total of 32 participants were tested. The sample (27 female, 4 left-handed, 19–31 years, mean age: 21.46 years) consisted of students from the University of Trier. All of the participants gave written informed consent prior to their participation.

### Apparatus and stimuli

The participants were tested in a dark, sound-attenuated laboratory. A touchpad (7’, resolution: 1680 × 1050 pixels; PPI: 265), operated with the corresponding touch stylus was attached to the participant’s left forearm. On the back of the touchpad, five tactors (Model C-2, Engineering Acoustic, Inc.; 3 cm in diameter, centrally located skin contactor of 0.76 cm) were attached and used to present the vibrotactile stimuli (~ 250 Hz, about 125 μm peak-to-peak amplitude) to the volar side of the forearm (see Fig. 1A). The tactors were arranged in a straight line with an approximate center-to-center distance of 3.5 cm. To avoid any distraction from the sound elicited by the tactors, the participants wore earplugs (noise reduction: 29 dB) and over-ear headphones through which brown noise (simultaneously-presented frequency distribution with higher intensities at lower frequencies, about 85 dB) was presented. The experiment was programmed with E-Prime 2.0, IBM SPSS statistics (Version 26) was used for data analyses.

### Procedure

Each trial consisted of the successive presentation of three vibrotactile stimuli. Both the duration and interstimulus interval (ISI) were fixed at 250 ms. After the third vibration, the participants indicated the perceived location of the last stimulus with the stylus on the touchpad. With their response, the participants completed the current trial and automatically started the next one.

Two different trial types were realized: For consistent motion trials, the three tactile stimuli implied consistent directional motion, that is, the stimuli were presented adjacent to each other translating in a consistent direction on every trial; for the inconsistent motion trials, the locations were selected randomly without replacement with the restriction that consistent motion trials never occurred. All the trials ended on the middle three tactors locations (B, C or D, see Fig. 1B). For a consistent motion in the proximal direction (i.e., towards the elbow), the central location C (sequence: E–D–C) or the outer location B (sequence: D–C–B) were used as the relevant locations. For those trials indicating motion in the distal direction (i.e., toward the wrist), the central location C (sequence: (A–B–C) or the outer location D (sequence: B–C–D) were used.

Crucially, three different task settings with different trial type probabilities were designed, and split into four different experimental blocks to present to each participant. In the first task setting, only consistent motion trials were presented (100% consistent motion trials). In a second task setting, only inconsistent motion trials were presented (100% inconsistent motion trials). In a third task setting, both trial types were mixed (50% consistent and 50% inconsistent motion trials). As the number of trials per trial type across task settings were kept identical to allow for better comparison, the mixed task setting had twice as many trials compared to the other two. Yet, in order to keep the length of the experimental blocks comparable, the mixed task setting was split in two experimental blocks and presented to each participant twice, so that every participant worked through four experimental blocks. The sequence of the experimental blocks was fixed, (100% consistent motion trials–50% consistent and inconsistent motion trials [part 1]–100% inconsistent motion trials–50% consistent and inconsistent motion trials [part 2]), yet, with which experimental block participants started was counterbalanced across participants using Latin square rules to prevent any effects of fatigue or related to systematically influence the data.

At the start of each block, the participants completed eight practice trials (trial types were selected from the first experimental block for this participant). This was followed by 64 experimental trials per experimental block. Overall participants worked through 256 experimental trials following the experimental design of 2 (trial type: consistent motion vs. inconsistent motion) × 2 (experimental condition: 50%—both trial types mixed vs. 100%—only one trial type) × 2 (relative location: central vs. outer) × 2 (direction: left-to-right vs. right-to-left) × 16 (repetitions).

### Design, data-preparation and analysis

The participants were tested in a 2 × 2 × 2 × 2 design with the four within-participants factors: *trial type* (consistent vs. inconsistent motion)*, experimental condition* (50%—both trial types mixed vs. 100%—only one trial type), *relative location* (central vs. outer), and *direction* (left-to-right vs. right-to-left). Absolute x- and y-axis scores in pixels were obtained for each trial.

In a first step, any potential effect of experimental condition on the inconsistent motion trials was analyzed. The inconsistent motion trials are taken as the baseline condition to account for general, motion independent localization biases (e.g., the centering bias; Brooks et al., 2019; Nelson et al., 2019—further influenced by intensity, e.g., Steenbergen et al., 2014; head orientation biases, Ho & Spence, 2007, anchoring by landmarks such as elbow or wrist, e.g., Cholewiak & Collins, 2003). The inconsistent motion trials are necessary to calculate the shift scores to investigate the existence of RM in touch (for similar approaches, see Merz et al., 2020, 2022). It was assumed that task setting had no influence on the inconsistent motion trials, as motion signals in inconsistent motion trials are not in one consistent direction, therefore not systematically influencing perception in one systematic direction. Therefore, in a pre-analysis of only the inconsistent motion trials, a 3 actual tactor location^1^ (tactor location B vs. C vs. D) × 2 experimental condition (50%—inconsistent motion vs. 100%—inconsistent motion) ANOVA with absolute x-axis scores (with higher values indicating a response more to the right on the touchpad) as the dependent variable was conducted (for a visualization of the results, see Fig. 1C). As expected, a main effect of actual tactor location was observed, *F*(2, 62) = 162.67, *p* < 0.001, η_p_^2^ = 0.840, yet, crucially, no main effect of experimental condition, *F*(1, 31) = 2.34, *p* = 0.136, η_p_^2^ = 0.070; nor any interaction between the two factors, *F*(2, 62) = 0.936, *p* = 0.398, η_p_^2^ = 0.126, were observed. This indicates that experimental condition, more precisely, trial type probabilities did not influence perception of inconsistent motion trials, as expected.

In a second step, the mean of the inconsistent motion trials was used as the baseline against which the results of the consistent motion trials were compared. That is, the dependent variable, the shift score, was computed as the difference of the x-axis scores in pixels between the location estimation of consistent motion trials and the (mean of the) inconsistent motion trials, for each of the eight combinations of experimental condition, direction and location separately. The shift scores were computed in such a way that a positive value indicates a shift in the direction of the consistent motion (relative to the inconsistent motion trials), while a negative value indicates a shift in the direction opposite to that of consistent motion. For example, a positive shift (forward shift) indicates a mean localization closer to the elbow (the left side of the tablet) for the right-to-left consistent motion trials as compared to the inconsistent motion trials. All data and experimental code are publicly available via OSF: https://osf.io/c4typ/?view_only=836b93c691d145118e3ead078eef2911.

## Results

A 2 (*experimental condition*: 50%—consistent motion vs. 100%—consistent motion) × 2 (*location:* central vs. outer) × 2 (*direction:* left-to-right vs. right-to-left) ANOVA with shift scores as dependent variable was computed. Crucially, the main effect of experimental condition was observed, *F*(1, 31) = 4.466, *p* = 0.043, η_p_^2^ = 0.126 (for a visualization, see Fig. 1D). That is, for the consistent motion trials in the mixed condition (50% consistent motion trials, 50% inconsistent motion trials), a clear forward shift was observed (40.29 pixel), *t*(31) = 3.51, *p* = 0.001, *d* = 0.62, yet, when only consistent motion trials were presented (100% consistent motion trials), no shift was observed (9.25 pixel), *t*(31) = 0.618, *p* = 0.541 (t-tests are comparisons against zero). None of the other main effects, nor any of the interactions, were significant, *F*s < 1.71, *p*s > 0.201.

## Discussion

The results indicate a clear effect of variations in the probability of different trial types on the perception of consistent motion trials, with the observation of a strong forward shift, that is, RM for the mixed condition. This is in line with previous research observing the RM phenomenon for consistent motion trials in touch when inconsistent motion trials are mixed (Merz et al., 2019a, 2019b, 2022). Yet, the non-existence of the RM effect for the experimental block in which only consistent motion trials were presented is in line with the study by Macauda et al. (2018) that failed to evidence a tactile analogue of RM. The present results are able to account for these previously contrasting findings regarding RM in touch by calling for the probability of different trial types to be taken into account.

The present study is able to resolve an apparent inconsistency in the tactile Representational Momentum literature by analysing the influence of trial type probability. Yet, the central question is, why does the inclusion / exclusion of inconsistent motion trials within the same experimental block have such a strong influence on the perception / localization of dynamic, consistently-moving stimuli? The consistent motion trials and the inconsistent motion trials differ in two key regards. First, whereas the three vibrations for the consistent motion trials are successive vibrations in one consistent direction, the inconsistent motion trials typically change direction between successive vibrations. Second, for the consistent motion trials, the vibrations are presented successively spatially adjacent to one another, the vibration for the inconsistent motion trials are spatially not systematically adjacent, therefore the distance from one vibration to the next could be much longer. With timing parameters (vibration duration and interstimulus interval) identical for both trial types, this type of manipulation results in a faster average speed for the inconsistent motion trials (because of the possibility of successive activation of nonadjacent tractors, e.g., “E – B”). Therefore, in a task setting with both trial types mixed, compared to one task setting with only consistent motion trials, the speed profile is much faster, which possibly might underlie the differing results. In fact, on a theoretical level, we recently proposed the speed prior account to explain many perceptual biases for dynamic objects on the basis of different speed expectations, including the RM phenomenon (Merz et al., 2022).

Following the speed prior idea (Merz et al., 2022), the RM phenomenon originates from the difference between the actual stimulus speed perceived by the sensory system (sensory input) and an expectation about the speed of the stimulus (prior). Both sources of information are then combined to inform the final percept, likely weighted by their relative uncertainty (for detailed discussion about the possible combination of sensory input and prior expectations, see e.g., Goldreich, 2007; Körding & Wolpert, 2004; Stocker & Simoncelli, 2006). In the condition with consistent and inconsistent trials mixed, the overall speed profile is much faster, therefore resulting in a change of the speed expectation compared to the experimental condition with only consistent motion trials presented. With perceived speed reflecting the combination of actual speed (sensory input) and speed expectation (prior), difference in speed expectations should result in differences in perceived speed, subsequently resulting in changes of perceived final location for identical stimuli, as observed in the present study. In case that the notion of different experimental conditions resulting in different speed expectation is true, as for example proposed by the speed prior account (similarly, see Hubbard, 1994), other expectation manipulations might be able to induce similar results as already shown in the literature (Reed & Vinson, 1996; Vinson & Reed, 2002). Yet, the speed prior account is not the only existing theory that may account for the present results, as already existing accounts could likely explain these results by proposing top-down/ expectation influences (e.g., Hubbard, 1995; Jancke & Erlhagen, 2010; for an overview, see Hubbard, 2010). Therefore, the existing theories need to be extended and refined to develop more precise predictions regarding the influence of expectations in order to be differentiated in future research.

The present results are likely somewhat surprising to those researchers who are more familiar with evidence from the literature on visual representational momentum. In fact, in vision, with our experimental set-up consisting of a consistent motion sequence with a stimulus duration of 250 ms and an ISI of 250 ms, it would clearly be expected that Representational Momentum would be observed when only consistently moving stimuli are presented (e.g., Freyd & Finke, 1984), and that it would likely decrease with other trial types mixed (e.g., effects of predictability / change on Representational Momentum: Kelly & Freyd, 1987; Kerzel, 2002). Yet, the reverse pattern was observed in the present study with tactile stimulation. The question arises as to how these differing results (in fact, the prediction in vision can be experimentally observed, Merz et al., 2023b) can be accounted for. Of course, it is possible to propose differing mechanisms underlying motion perception for each of the senses. Yet, our own preferred explanation would be to explain the divide with changes of speed expectations (e.g., the speed prior account, Merz et al., 2022) and proposing different speed expectations and adaptations thereof in the different sensory modalities, as the stimulation perceived in vision and touch are likely very different (e.g., extend / range of motion trajectory restricted by natural landmarks / finite space along the skin in touch compared to more unrestricted motion possibilities in vision; tactile stimulation takes place at the body surface, whereas visual stimulation takes place at as well as away from the body surface; sensory acuity is very different across the two senses; for discussion, see Pei & Bensmaia, 2014). Alternatively, differences in motion trajectory predictability might be a driving factor underlying the observed data pattern, yet, future research needs to be conducted to more appropriately tackle this question.

In summary, the present study was able to resolve seemingly contrasting findings conconcerning the existence of tactile RM. By systematically exploring the influence of varying probabilities of different trial types, our study indicates a clear influence of other trial types mixed in the current experimental task setting. This calls for the importance for those researchers interested in human (motion) perception to be mindful about the trial types added (or discarded) for any experiments in general, as these could result in changes of (speed) expectations.

## Data Availability

Raw data, experimental files and important analyses scripts are publicly available at OSF: https://osf.io/c4typ/?view_only=836b93c691d145118e3ead078eef2911. The experiment was not preregistered.

## Footnotes

1 Note that the two factors direction and relative location from the overall design of the experiments were combined in only three different final locations, as the factor direction does not have informational value for the inconsistent motion trials, as for this trial type, the central objective was to not have a consistent direction information. Therefore, the final target location for the condition combination “right-to-left – outer” corresponds to tactor location B, the final target location for both, the “right-to-left – central” and “left-to-right – central”, corresponds to tactor location C, and the final target location for the “left-to-right – outer” corresponds to tactor location D.

## References

[CR1] Brooks J, Seizova-Cajic T, Taylor JL (2019). Biases in tactile localization by pointing: Compression for weak stimuli and centering for distributions of stimuli. Journal of Neurophysiology.

[CR2] Cholewiak RW, Collins AA (2003). Vibrotactile localization on the arm: Effects of place, space, and age. Perception & Psychophysics.

[CR3] Faul F, Erdfelder E, Lang A-G, Buchner A (2007). G*power 3: A flexible statistical power analysis program for the social, behavioral, and biomedical sciences. Behavior Research Methods.

[CR4] Freyd JJ, Finke RA (1984). Representational momentum. Journal of Experimental Psychology: Learning, Memory, and Cognition.

[CR5] Getzmann S, Lewald J (2007). Localization of moving sound. Perception & Psychophysics.

[CR6] Getzmann S, Lewald J, Guski R (2004). Representational momentum in spatial hearing. Perception.

[CR7] Goldreich D (2007). A Bayesian perceptual model replicates the cutaneous rabbit and other tactile spatiotemporal illusions. PLoS ONE.

[CR8] Hayes AE, Freyd JJ (2002). Representational momentum when attention is divided. Visual Cognition.

[CR9] Ho C, Spence C (2007). Head orientation biases tactile localization. Brain Research.

[CR10] Hubbard TL (1994). Judged displacement: A modular process?. The American Journal of Psychology.

[CR11] Hubbard TL (1995). Environmental invariants in the representation of motion: Implied dynamics and representational momentum, gravity, friction, and centripetal force. Psychonomic Bulletin & Review.

[CR12] Hubbard TL (2005). Representational momentum and related displacements in spatial memory: A review of the findings. Psychonomic Bulletin & Review.

[CR13] Hubbard TL, Nijhawan R, Khurana B (2010). Approaches to representational momentum: Theories and models. Space and time in perception and action.

[CR14] Hubbard TL, Hubbard TL (2018). Influences on representational momentum. Spatial biases in perception and cognition.

[CR15] Hubbard TL, Ruppel SE (2014). An effect of contrast and luminance on visual representational momentum for location. Perception.

[CR16] Jancke D, Erlhagen W, Nijhawan R, Khurana B (2010). Bridging the gap: A model of common neural mechanisms underlying the Fröhlich effect, the flash-lag effect, and the representational momentum effect. Space and time in perception and action.

[CR17] Kelly MH, Freyd JJ (1987). Explorations of representational momentum. Cognitive Psychology.

[CR18] Kerzel D (2002). A matter of design: No representational momentum without predictability. Visual Cognition.

[CR19] Körding KP, Wolpert DM (2004). Bayesian integration in sensorimotor learning. Nature.

[CR20] Macauda G, Lenggenhager B, Meier R, Essick G, Brugger P (2018). Tactile motion lacks momentum. Psychological Research.

[CR21] Mancini F, Longo MR, Iannetti GD, Haggard P (2011). A supramodal representation of the body surface. Neuropsychologia.

[CR22] Merz S, Deller J, Meyerhoff HS, Spence C, Frings C (2019). The contradictory influence of velocity: Representational momentum in the tactile modality. Journal of Neurophysiology.

[CR23] Merz, S., Frings, C., & Spence, C. (2023a). Motion perception in touch: Resolving contradictory findings by focusing on experimental context. *Psychological Research* (under review). 10.1007/s00426-023-01849-1 PMC10805958 37369933

[CR24] Merz S, Meyerhoff HS, Frings C, Spence C (2020). Representational momentum in vision and touch: Visual motion information biases tactile spatial localization. Attention, Perception & Psychophysics.

[CR25] Merz S, Meyerhoff HS, Spence C, Frings C (2019). Implied tactile motion: Localizing dynamic stimulations on the skin. Attention, Perception & Psychophysics.

[CR26] Merz S, Soballa P, Spence C, Frings C (2022). The speed prior account: A new theory to explain multiple phenomena regarding dynamic information. Journal of Experimental Psychology: General.

[CR27] Merz, S., Spence, C., & Frings, C. (2023b). Need for (expected) speed: Exploring the effect of trial type probabilitiy on representational momentum. *Attention, Perception, & Psychophysics* (under review). 10.3758/s13414-023-02796-0 PMC10600037 37821746

[CR28] Nelson JS, Baud-Bovy G, Smeets JBJ, Brenner E (2019). Accuracy of Intercepting Moving Tactile Targets. Perception.

[CR29] Pei Y-C, Bensmaia SJ (2014). The neural basis of tactile motion perception. Journal of Neurophysiology.

[CR30] Pei Y-C, Hsiao SS, Craig JC, Bensmaia SJ (2011). Neural mechanisms of tactile motion integration in somatosensory cortex. Neuron.

[CR31] Reed CL, Vinson NG (1996). Conceptual effects on representational momentum. Journal of Experimental Psychology: Human Perception and Performance.

[CR32] Schmiedchen K, Freigang C, Rübsamen R, Richter N (2013). A comparison of visual and auditory representational momentum in spatial tasks. Attention, Perception & Psychophysics.

[CR33] Steenbergen P, Buitenweg JR, Trojan J, Veltink PH (2014). Tactile localization depends on stimulus intensity. Experimental Brain Research.

[CR34] Stocker AA, Simoncelli EP (2006). Noise characteristics and prior expectations in human visual speed perception. Nature Neuroscience.

[CR35] Vinson NG, Reed CL (2002). Sources of object-specific effects in representational momentum. Visual Cognition.

[CR36] Whitsel BL, Franzen O, Dreyer DA, Hollins M, Young M, Essick GK, Wong C (1986). Dependence of subjective traverse length on velocity of moving tactile stimuli. Somatosensory Research.

